# Supplementary material for: Amorphous (lysine)2PbI2 layer enhanced perovskite photovoltaics
Source: Nat Commun. 2024 Aug 17;15:7085. doi: 10.1038/s41467-024-51551-y (PMC11330473; doi:10.1038/s41467-024-51551-y)
Supplement: Supplementary file 1 — Supplementary Information [file 41467_2024_51551_MOESM1_ESM.pdf]

Supplementary Information for

Amorphous (lysine)$_2$PbI$_2$ Layer Enhanced Perovskite Photovoltaics

Yehui Wen[1,2, †], Tianchi Zhang[1,2, †], Xingtao Wang [3,†], Tiantian Liu [4,†], Yu Wang [5], Rui Zhang [5], Miao Kan [6], Li Wan [7], Weihua Ning [2,*], Yong Wang [1, *], Deren Yang[1, *]

1. State Key Laboratory of Silicon and Advanced Semiconductor Materials and School of Materials Science and Engineering, Hangzhou Global Scientific and Technological Innovation Center, Zhejiang University, Hangzhou, Zhejiang 310027, P. R. China.

2. Institute of Functional Nano & Soft Materials, Joint International Research Laboratory of Carbon-Based Functional Materials and Devices, Soochow University, Suzhou 215123, P. R. China.

3. Huaneng Clean Energy Research Institute, Beijing 102209, China

4. School of Chemistry and Chemical Engineering, Xi'an University of Architecture and Technology, Xi'an, 710055, China

5. Department of Physics, Chemistry, and Biology (IFM), Linköping University, Linköping 58183, Sweden

6. School of Chemistry and Molecular Engineering East China University of Science and Technology130 Meilong Road, Shanghai 200237, China

7. Max Planck Institute of Microstructure Physics, 06120 Halle, Germany

**Corresponding author. Email: mseyang@zju.edu.cn; yonwa12@zju.edu.cn; whning@suda.edu.cn.*

† These authors contributed equally to this work.

This file contains

Supplementary Figure. S1 to S27

Supplementary Tables S1 to S2

Supplementary Reference 1-3

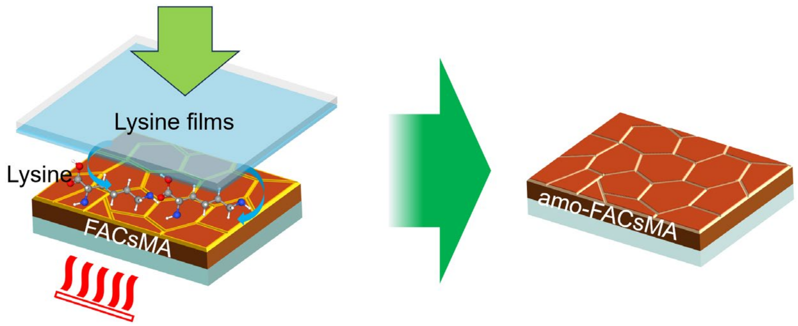

Supplementary Fig. S1 Schematic diagram illustrating amo-FACsMA film prepared by the solid-state diffusion of lysine and the subsequently solid-state reaction of lysine and PbI_2 resides.

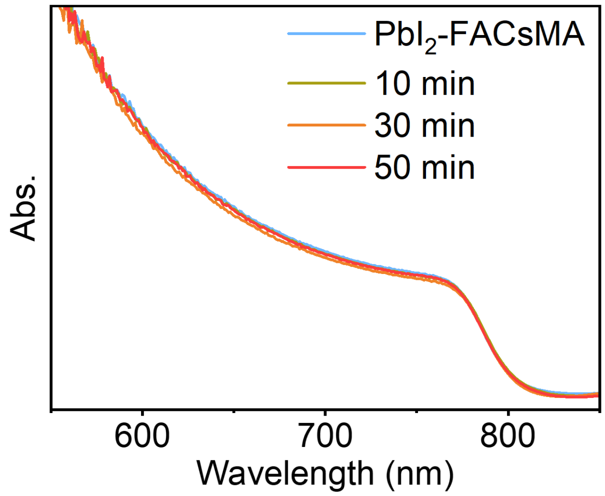

Supplementary Fig. S2 UV-vis spectra for perovskite films with different lysine diffusion period, abs, absorption.

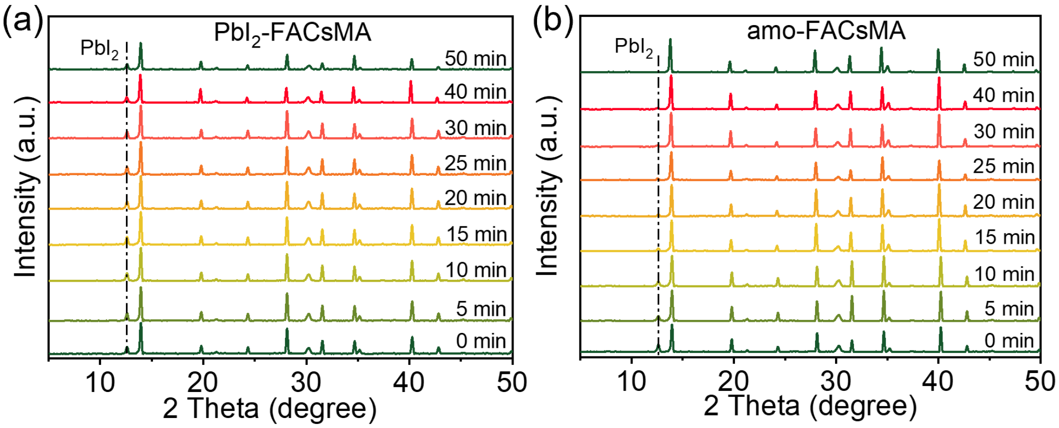

Supplementary Fig. S3 Effect of lysine diffusion on the crystal structure of perovskite. (a) XRD patterns for PbI_2-FACsMA film covered by a piece of clean glass. (b) XRD patterns for perovskite films with different lysine diffusion period.

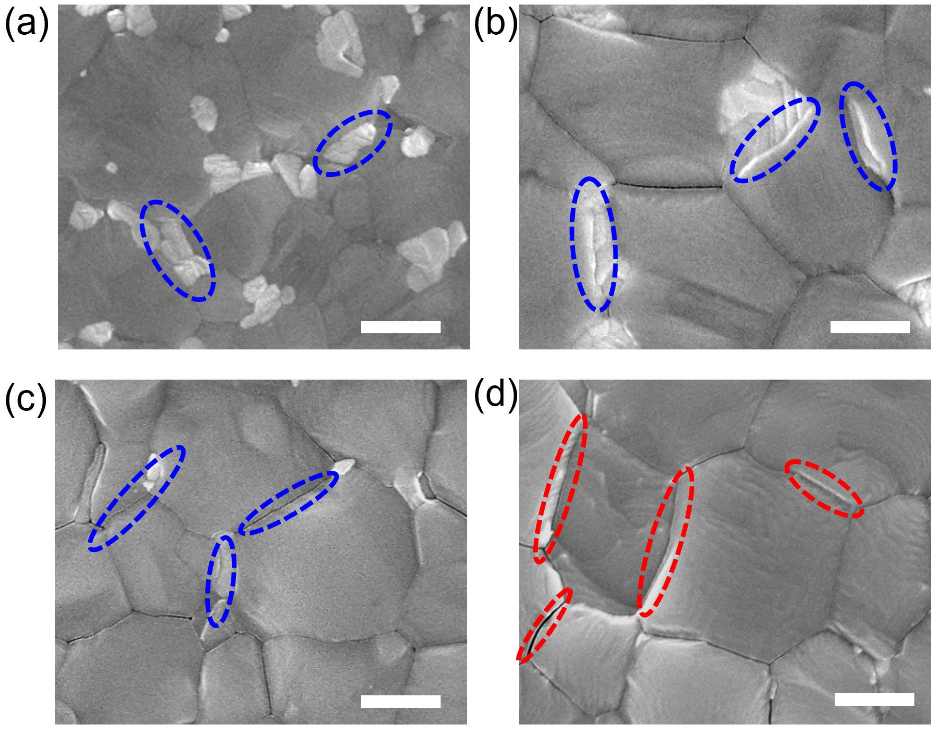

Supplementary Fig. S4 High-resolution SEM morphologies for perovskite films with different lysine diffusion period. (a) 0 min, (b) 10 min, (c) 30 min, (d) 50 min. Scale bars represent 500 nm. Scale bars represent 500 nm. Scale bars represent 500 nm.

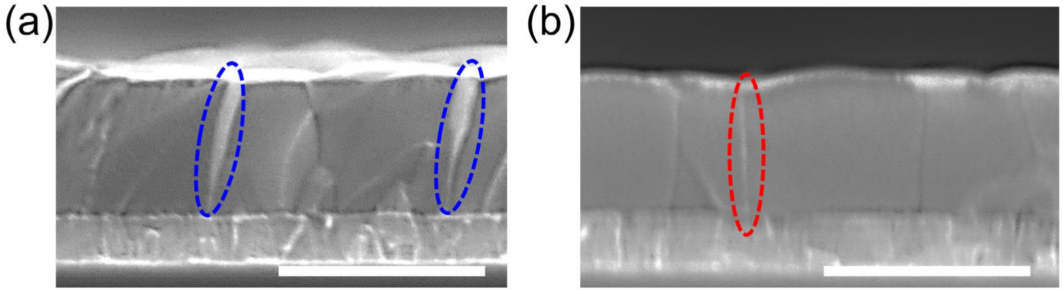

Supplementary Fig. S5 Zooming in on segments of the cross-sectional SEM images depicted in Figs 1b and 1c. (a) PbI_2-FACsMA; (b) amo-FACsMA, scale bars represent 1 μm.

The high-resolution cross-section morphologies in supplementary Fig. S5 show that a thick PbI_2 strips are interposed into the crystal grains in the PbI_2-FACsMA perovskite film, while a very thin layer is formed between the grains in the amo-FACsMA sample.

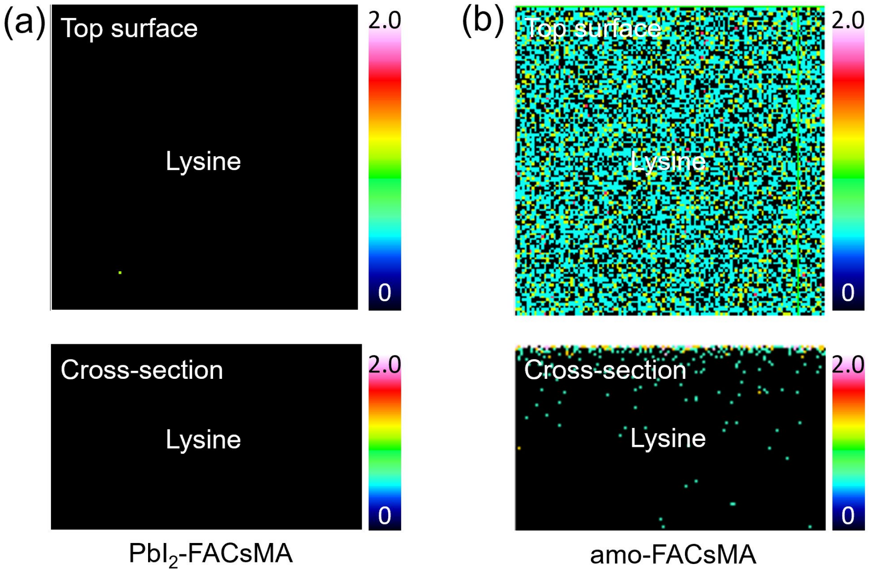

Supplementary Fig. S6 Lysine components analysis. Two-dimensional image (100 μm × 100 μm) of total depth accumulation (up), and cross-section image (down) in (a) PbI_2-FACsMA and (b) amo-FACsMA perovskite films.

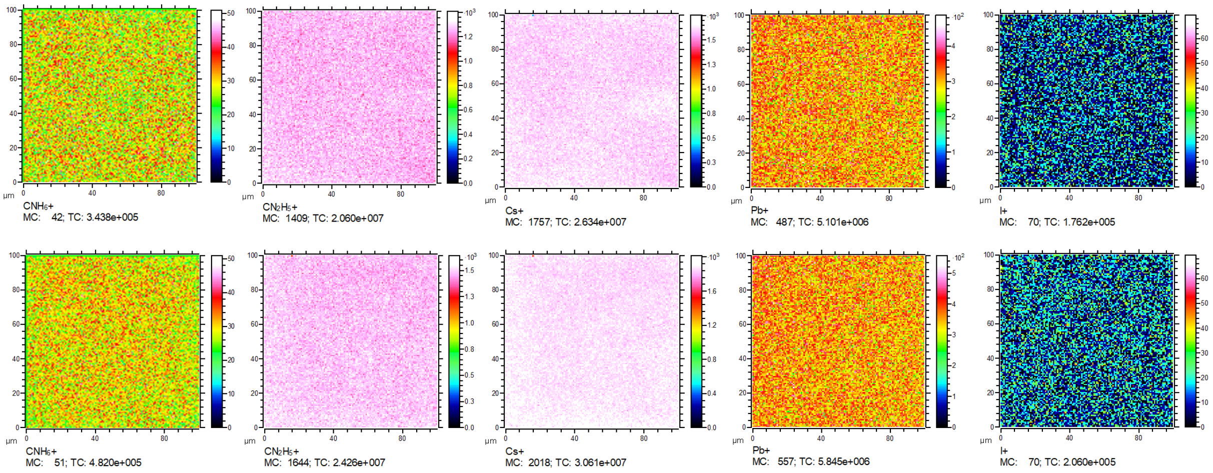

Supplementary Fig. S7 Composition distribution for PbI_2-FACsMA (up) and amo-FACsMA (down) perovskite films.

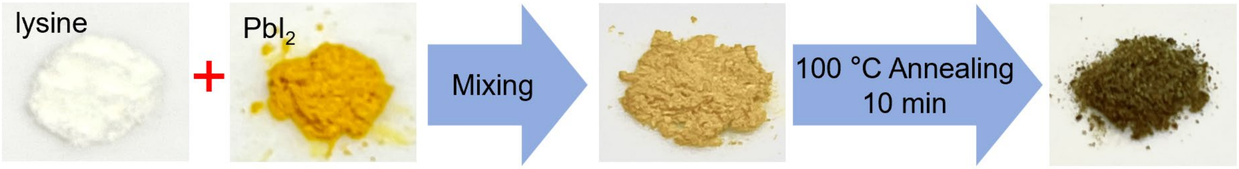

Supplementary Fig. S8 Illustration depicting the synthesis of $(lysine)_2PbI_2$.

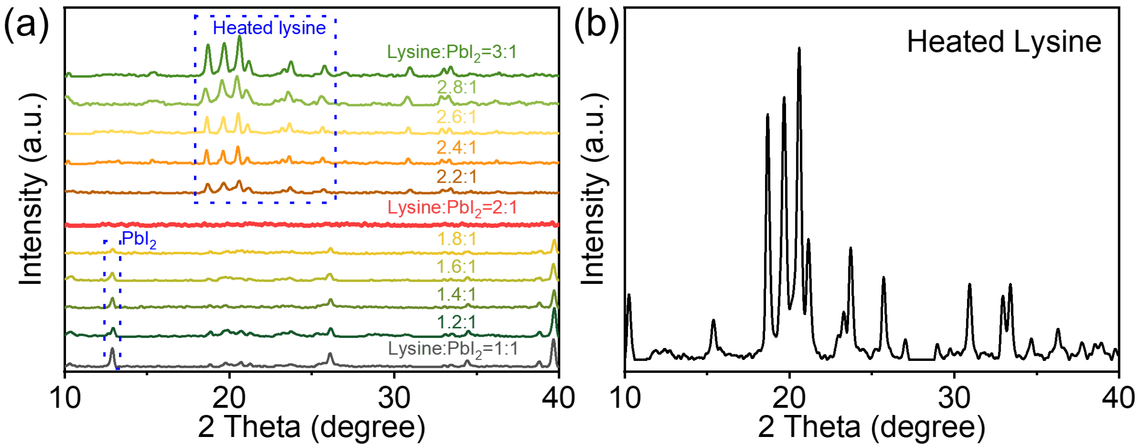

Supplementary Fig. S9 XRD patterns for the powders with different ratios of PbI_2: lysine.
(a) lysine-PbI_2 coordination powders, (b) heated lysine.

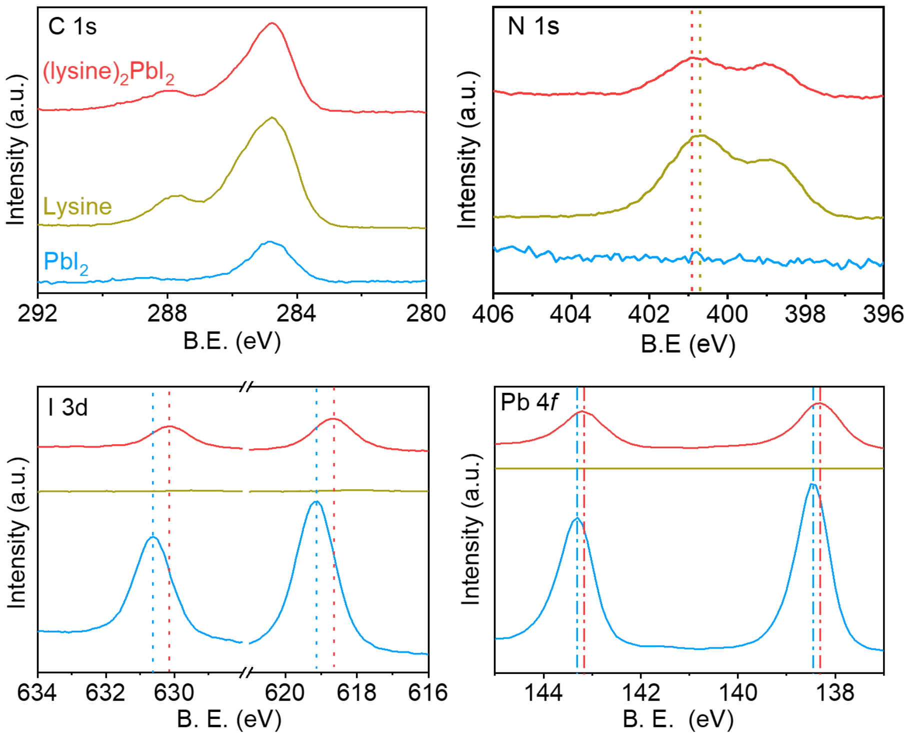

Supplementary Fig. S10 XPS results of PbI_2, lysine, and $(lysine)_2PbI_2$. High resolution (a) C 1s, (b) N 1s, (c) I 3d, and (d) Pb 4f core-level spectra.

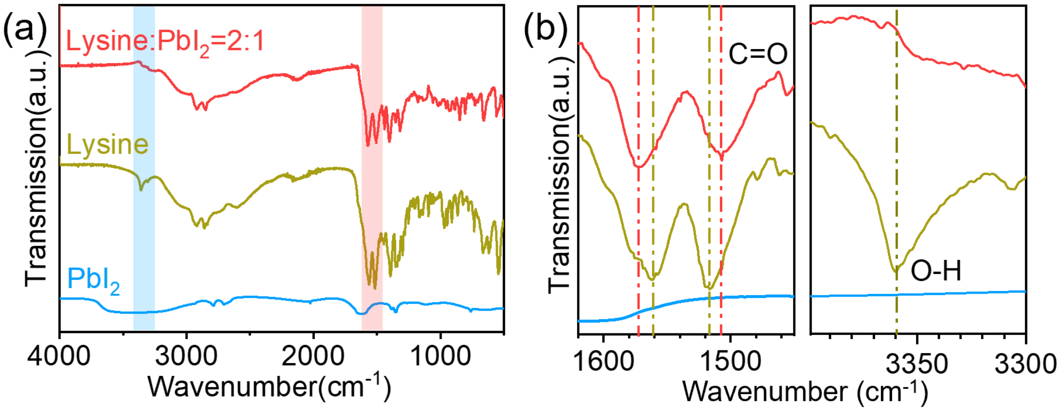

Supplementary Fig. S11 FTIR spectra for PbI_2, Lysine, $(Lysine)_2PbI_2$ **powders.** (a) FTIR spectra. (b) The local enlarged area in the (a).

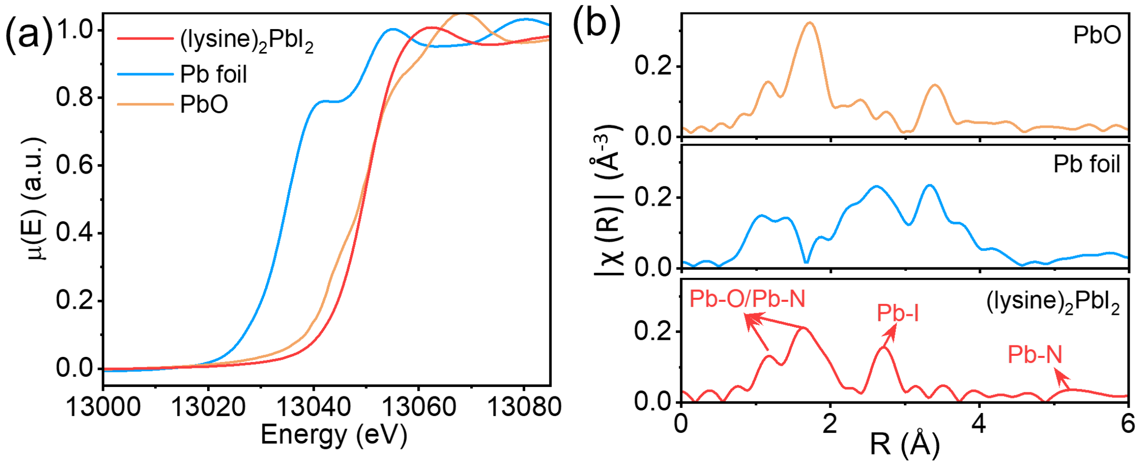

Supplementary Fig. S12 (a) Pb L_3-edge XANES spectra and (b) Fourier transformed EXAFS spectra for Pb foil, PbO, $lysine_2PbI_2$

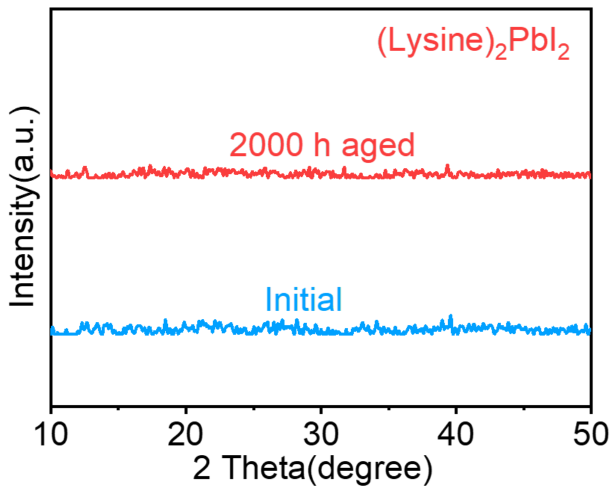

Supplementary Fig. S13 XRD patterns for the (lysine)$_2$PbI$_2$ after storing 2000 hours under dry condition.

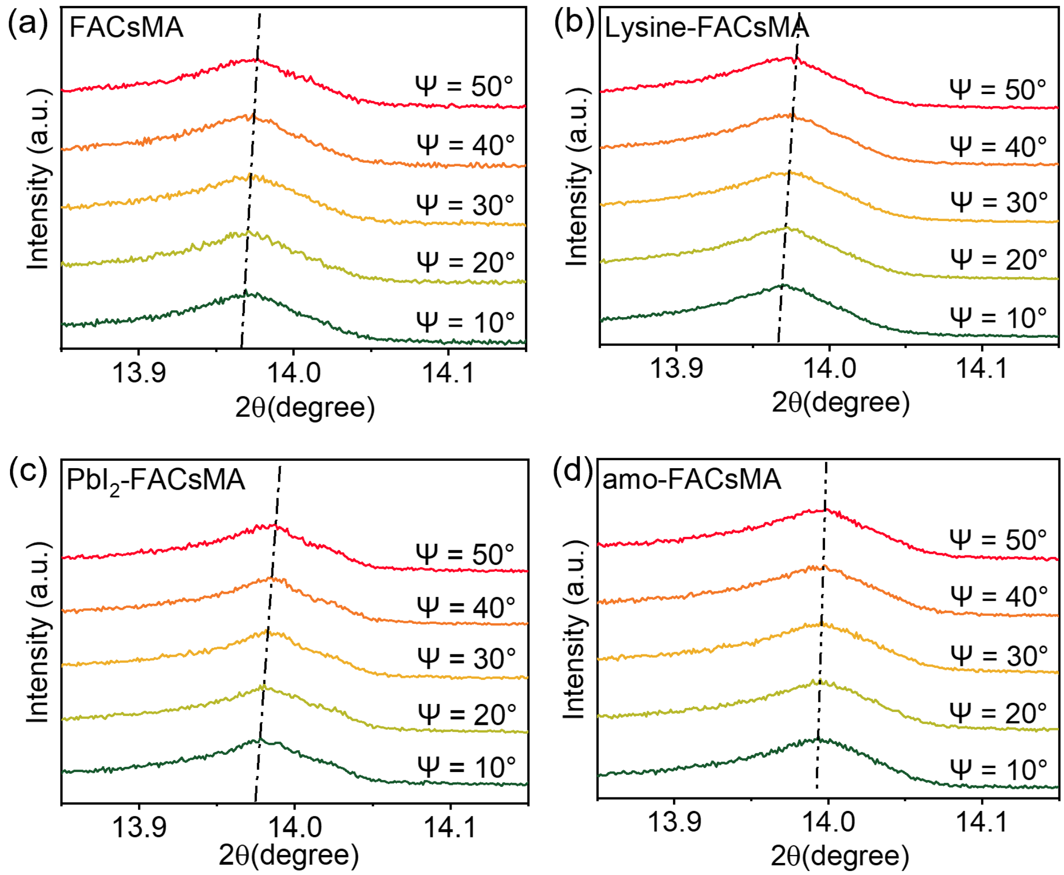

Supplementary Fig. S14 Depth-resolved GIXRD spectra at different Ψ angles (from 10° to 50°). (a) FACsMA, (b) lysine-FACsMA, (c) PbI_2-FACsMA, and (d) amo-FACsMA film.

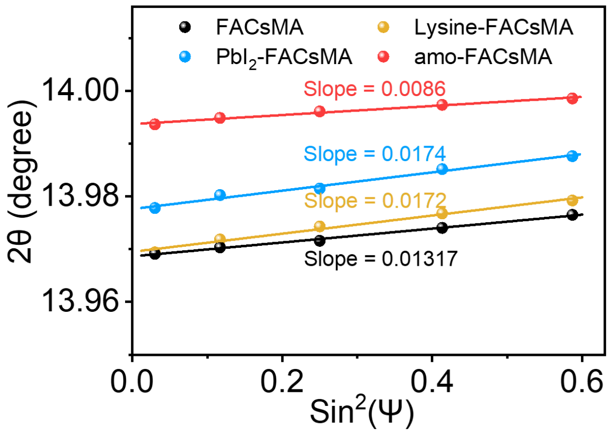

Supplementary Fig. S15 Linear fit of 2θ-sin2(Ψ) for perovskite films derived from GIXRD.

Herein, $FA_{0.85}MA_{0.1}Cs_{0.05}PbI_3$ is regard as reference sample (denoted as FACsMA), the FACsMA sample with lysine solely addition is denoted as is lysine-FACsMA. Generally, both PbI_2 and lysine can be regarded as crystal passivating materials. Firstly, we investigate the effect of different passivated materials on the lattice stress using Depth-dependent grazing incident XRD (GIXRD) measurements.[1] Based on the equation $\sigma = -\frac{E}{2(1+\nu)}\frac{\pi}{180}\cot\theta_0\frac{\partial(2\theta)}{\partial\sin^2\psi}$ (Where Ψ, E are the angles of the diffraction vector respective to the sample surface normal and the perovskite modulus, respectively). By varying the tilt angle Ψ from 10° to 50°, the intensity and position of the (001) peak within perovskites are traced. FACsMA film displays a positive slope of 0.01317 in the sin2Ψ-2θ plot, indicating stress in the spacing of its (100) crystal planes and the presence of residual tensile stress. However, with PbI_2 or lysine addition, the increased slope are observed on both PbI_2-FACsMA and lysine-FACsMA films, an indicative of increased lattice stress. Such increased lattice could be attributed to the strong ion-bonding effect of lysine molecules or mismatched lattice compatibility between PbI_2 and perovskite. In contrast, the amo-FACsMA film exhibits a significantly reduced slope, indicating the elimination of residual stress within the lattice. This result indicates that the amorphous $(lysine)_2PbI_2$ exhibits improved compatibility to

perovskite crystals, which is beneficial to alleviating the lattice stress. The reduced lattice stress not only favors enhancing stability but also reduces defects. [1-3]

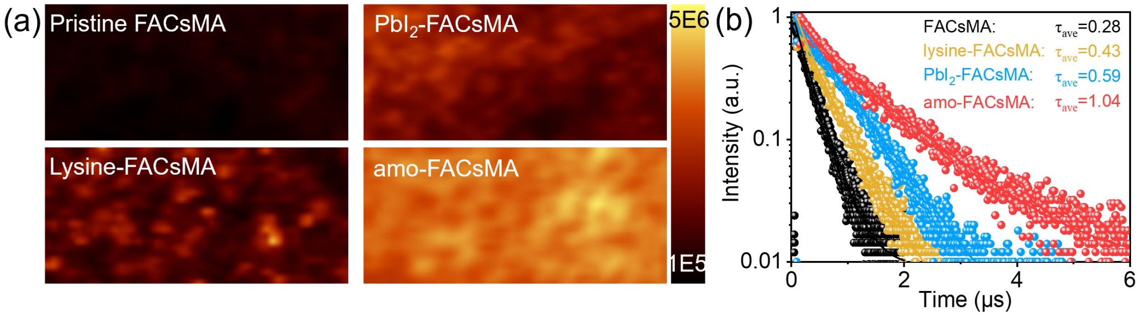

Supplementary Fig. S16 Fluorescence spectral characterization of perovskite films. (a) PL mapping results for FACsMA, lysine-FACsMA, PbI_2-FACsMA and amo-FACsMA films (area: 10×5 μm^2), respectively. (b) TRPL curves of FACsMA, lysine-FACsMA, PbI_2-FACsMA and amo-FACsMA films.

The time-resolved PL (TRPL) spectra in supplementary Fig. 16b shows that the PL lifetime (τ) of amo-FACsMA is much longer (1.04 μs) than that of pristine FACsMA, PbI_2-FACsMA (0.59 μs) and lysine-FACsMA (0.43 μs).

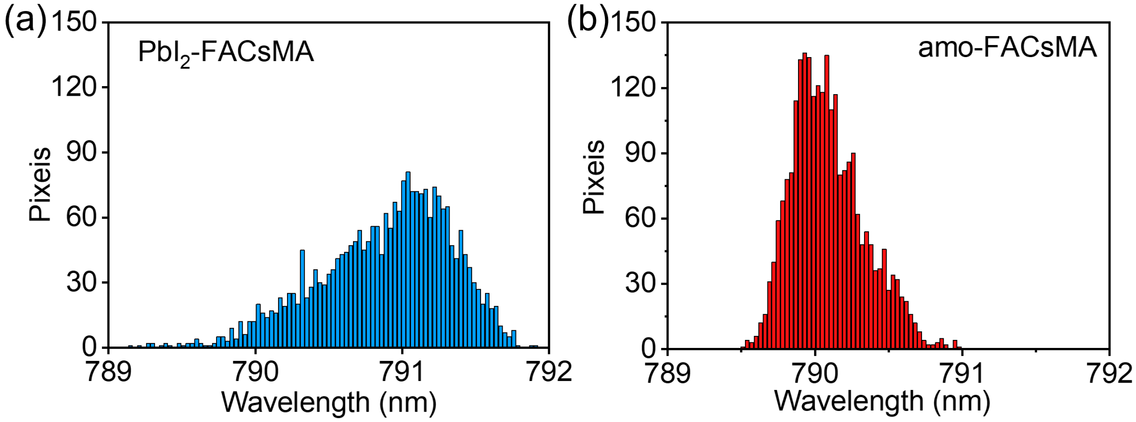

Supplementary Fig. S17 Statistics of PL luminescence obtained from Fig. S16a. (a) PbI_2-FACsMA and (b) amo-FACsMA films.

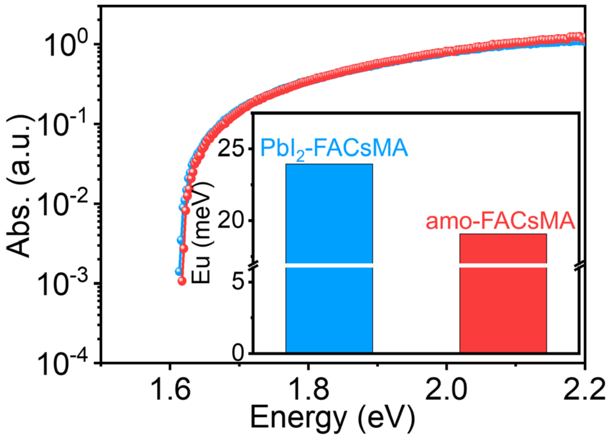

Supplementary Fig. S18 Effective absorption coefficient of the PbI_2-FACsMA and AMO-FACsMA films. The inset shows the average Urbach energies for these samples.

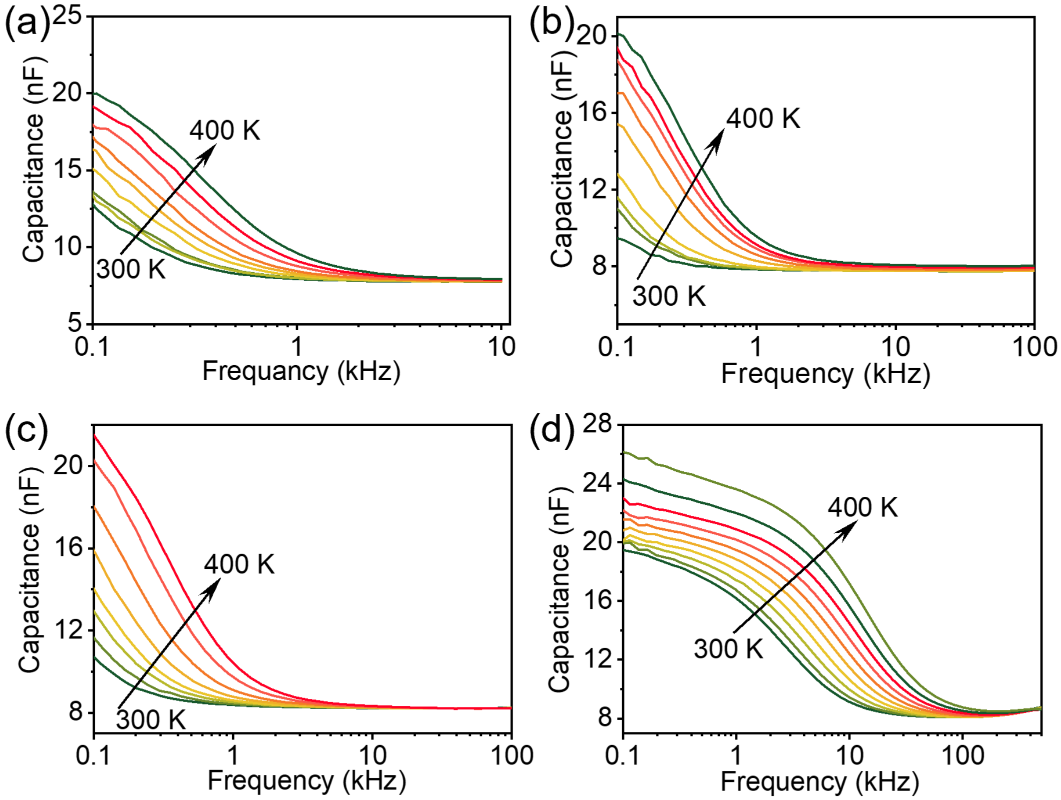

Supplementary Fig. S19 Temperature dependence of C–f plots. (a) FACsMA, (B) lysine-FACsMA, (c) PbI_2-FACsMA, and (d) amo-FACsMA devices.

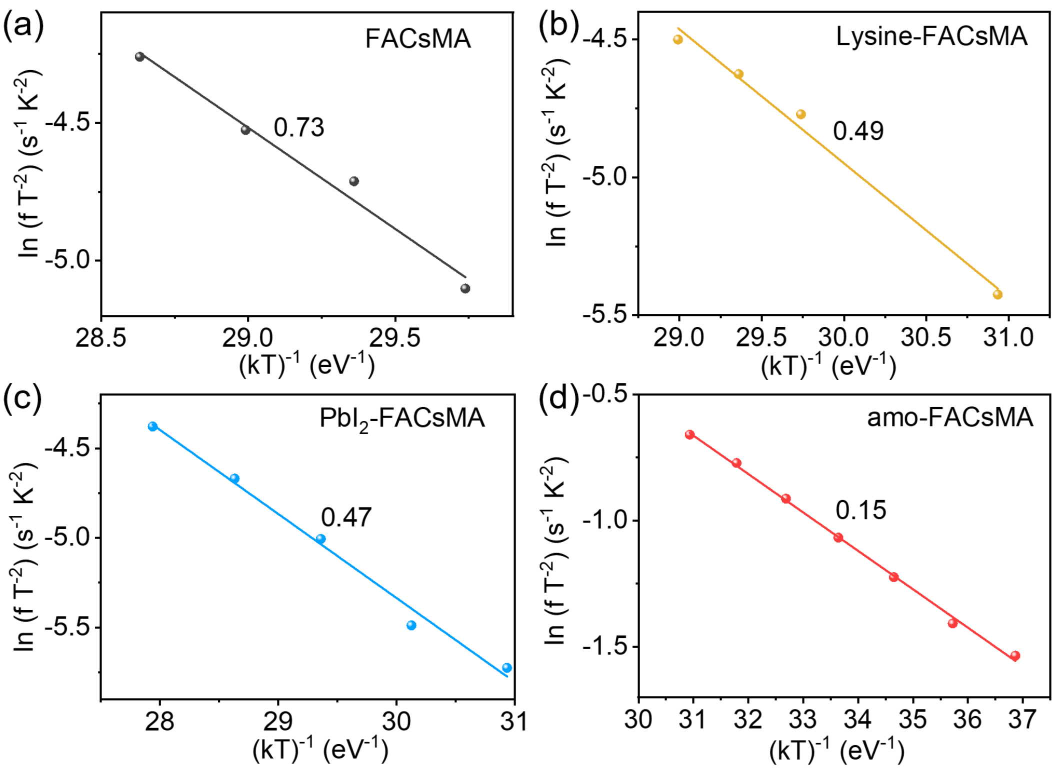

Supplementary Fig. S20 The characteristic frequencies deduced from temperature-dependent capacitance-frequency curves as shown in Fig. S19. (a) FACsMA, (b) lysine-FACsMA, (c) PbI_2-FACsMA, and (d) amo-FACsMA devices.

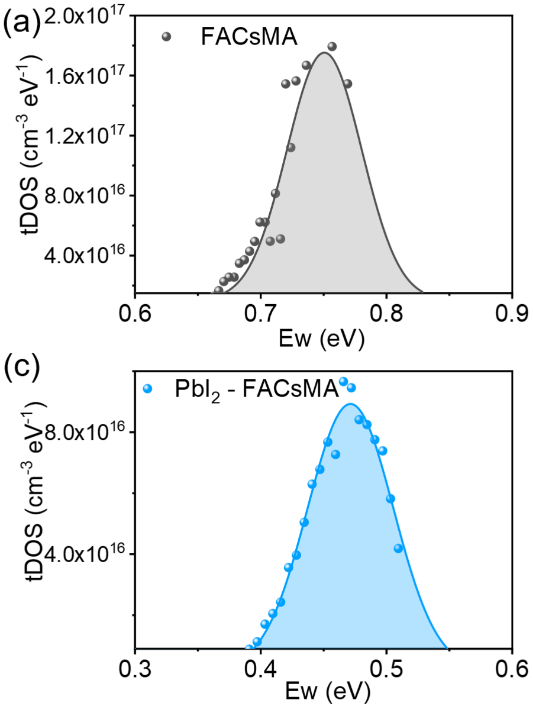

Supplementary Fig. S21 tDOS deduced from the room-temperature C–f plots. (a) FACsMA, (b) lysine-FACsMA, (c) PbI_2-FACsMA, and (d) amo-FACsMA devices.

Fig. 2c and Fig. S21 show the trap density deduced from the room-temperature C–f plots, giving an integrated trap density of 1.2×10^{16} cm^{-3} eV^{-1}, 7.4×10^{15} cm^{-3} eV^{-1}, 7.1×10^{15} cm^{-3} eV^{-1}, and 3.2×10^{15} cm^{-3} eV^{-1} for the FACsMA, lysine-FACsMA, PbI_2-FACsMA and amo-FACsMA, respectively.

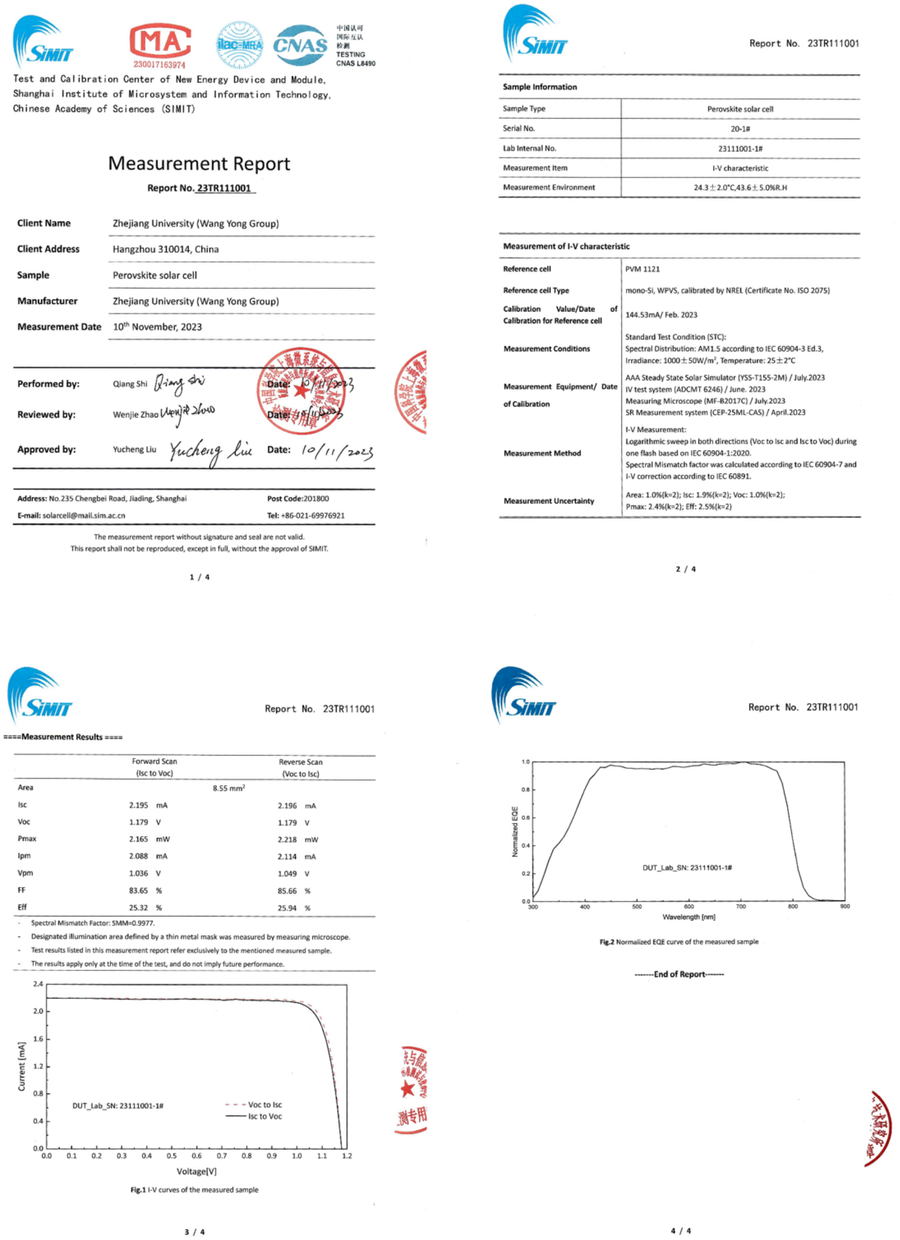

Measurement Report

Report No. 23TR111001

Client Name	Zhejiang University (Wang Yong Group)
Client Address	Hangzhou 310014, China
Sample	Perovskite solar cell
Manufacturer	Zhejiang University (Wang Yong Group)
Measurement Date	10th November, 2023

Performed by:	Qiang Shi	Date: 10/11/2023
Reviewed by:	Wenjie Zhao	Date: 10/11/2023
Approved by:	Yucheng Liu	Date: 10/11/2023

Address: No.235 Chengbei Road, Jiading, Shanghai Post Code:201800

E-mail: solarcell@mail.sim.ac.cn Tel: +86-021-69976921

The measurement report without signature and seal are not valid.

This report shall not be reproduced, except in full, without the approval of SIMIT.

1 / 4

Report No. 23TR111001

Sample Information

Sample Type	Perovskite solar cell
Serial No.	20-1#
Lab Internal No.	23111001-1#
Measurement Item	I-V characteristic
Measurement Environment	24.3±2.0°C,43.6±5.0%R.H

Measurement of I-V characteristic

Reference cell	PVM 1121
Reference cell Type	mono-Si, WPVS, calibrated by NREL (Certificate No. ISO 2075)
Calibration Value/Date of Calibration for Reference cell	144.53mA/ Feb. 2023
Measurement Conditions	Standard Test Condition (STC): Spectral Distribution: AM1.5 according to IEC 60904-3 Ed.3, Irradiance: 1000±50W/m², Temperature: 25±2°C
Measurement Equipment/ Date of Calibration	AAA Steady State Solar Simulator (YSS-T155-2M) / July.2023<br>IV test system (ADCMT 6246) / June. 2023<br>Measuring Microscope (MF-B2017C) / July.2023<br>SR Measurement system (CEP-25ML-CAS) / April.2023
Measurement Method	I-V Measurement:<br>Logarithmic sweep in both directions (Voc to Isc and Isc to Voc) during one flash based on IEC 60904-1:2020.<br>Spectral Mismatch factor was calculated according to IEC 60904-7 and I-V correction according to IEC 60891.
Measurement Uncertainty	Area: 1.0%(k=2); Isc: 1.9%(k=2); Voc: 1.0%(k=2);<br>Pmax: 2.4%(k=2); Eff: 2.5%(k=2)

2 / 4

Report No. 23TR111001

====Measurement Results ====

	Forward Scan (Isc to Voc)	Reverse Scan (Voc to Isc)
Area	8.55 mm²	
Isc	2.195 mA	2.196 mA
Voc	1.179 V	1.179 V
Pmax	2.165 mW	2.218 mW
Ipm	2.088 mA	2.114 mA
Vpm	1.036 V	1.049 V
FF	83.65 %	85.66 %
Eff	25.32 %	25.94 %

- Spectral Mismatch Factor: SMM=0.9977.
- Designated illumination area defined by a thin metal mask was measured by measuring microscope.
- Test results listed in this measurement report refer exclusively to the mentioned measured sample.
- The results apply only at the time of the test, and do not imply future performance.

Fig.1 I-V curves of the measured sample

3 / 4

Report No. 23TR111001

Fig.2 Normalized EQE curve of the measured sample

-------End of Report-------

4 / 4

Supplementary Fig. S22 Efficiency Certification Report. An independent PCE testing report of an amo-FACsMA based PSC device by an accredited PV center (Shanghai Institute of Microsystem and Information Technology, China) verified a PCE of 25.94% (reverse scan, a *I*sc of 2.196 mA, a *V*oc of 1.179 V, and an FF of 85.66%; forward scan, a *I*sc of 2.195 mA, a *V*oc of 1.179 V, and an FF of 83.65%). The cell was tested in air without encapsulation or protection during the testing process. Permission to use the logo was obtained from SIMIT.

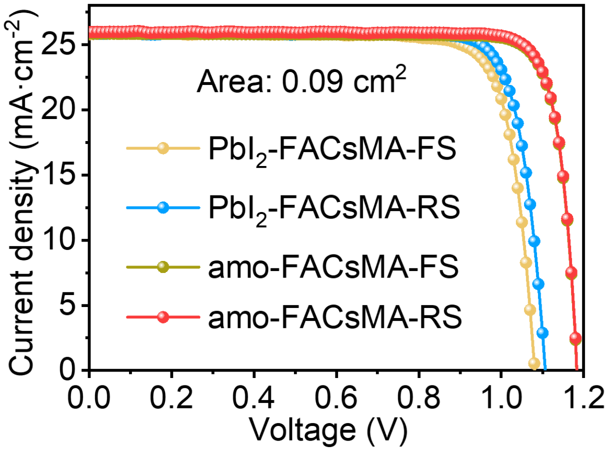

Supplementary Fig. S23 J-V curves for PSCs based on PbI_2-FACsMA and amo-FACsMA thin films in the forward and reverse scans (0.09 cm^2 active area).

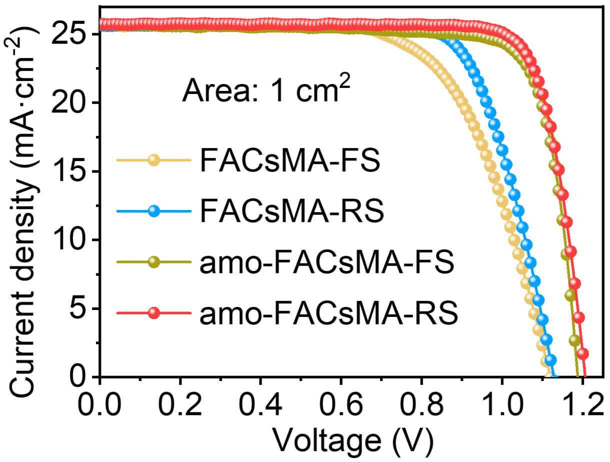

Supplementary Fig. S24 J-V curves for PSCs based on PbI_2-FACsMA and amo-FACsMA thin films in the forward and reverse scans (1 cm^2 active area).

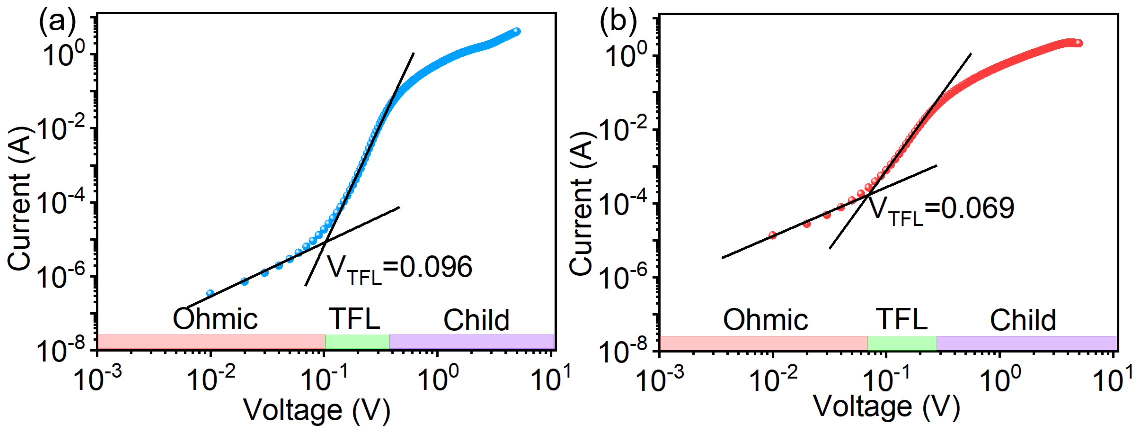

Supplementary Fig. S25 SCLC characteristics of the electron-only devices. (a) PbI_2-FACsMA, and (b) amo-FACsMA.

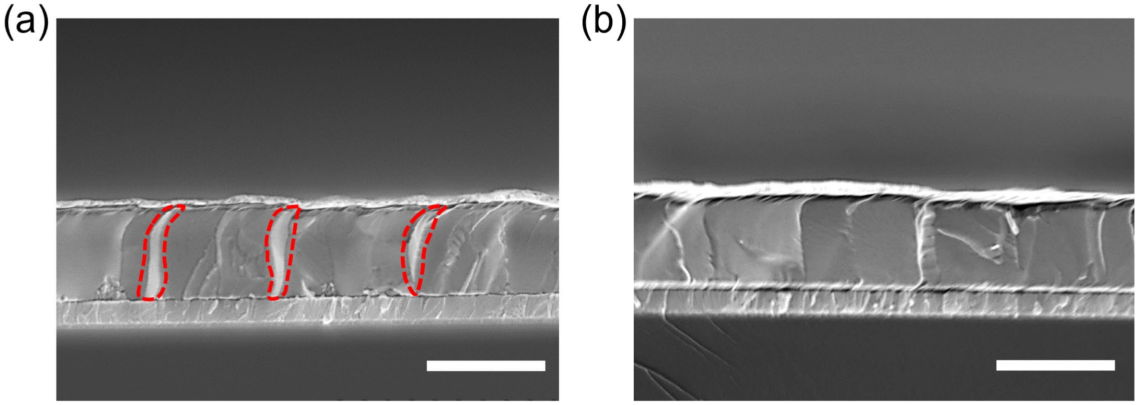

Supplementary Fig. S26 Initial cross-section images of PSCs before operational stability test. (a) PbI_2-FACsMA and (b) amo-FACsMA PSCs, scale bars represent 1 μm.

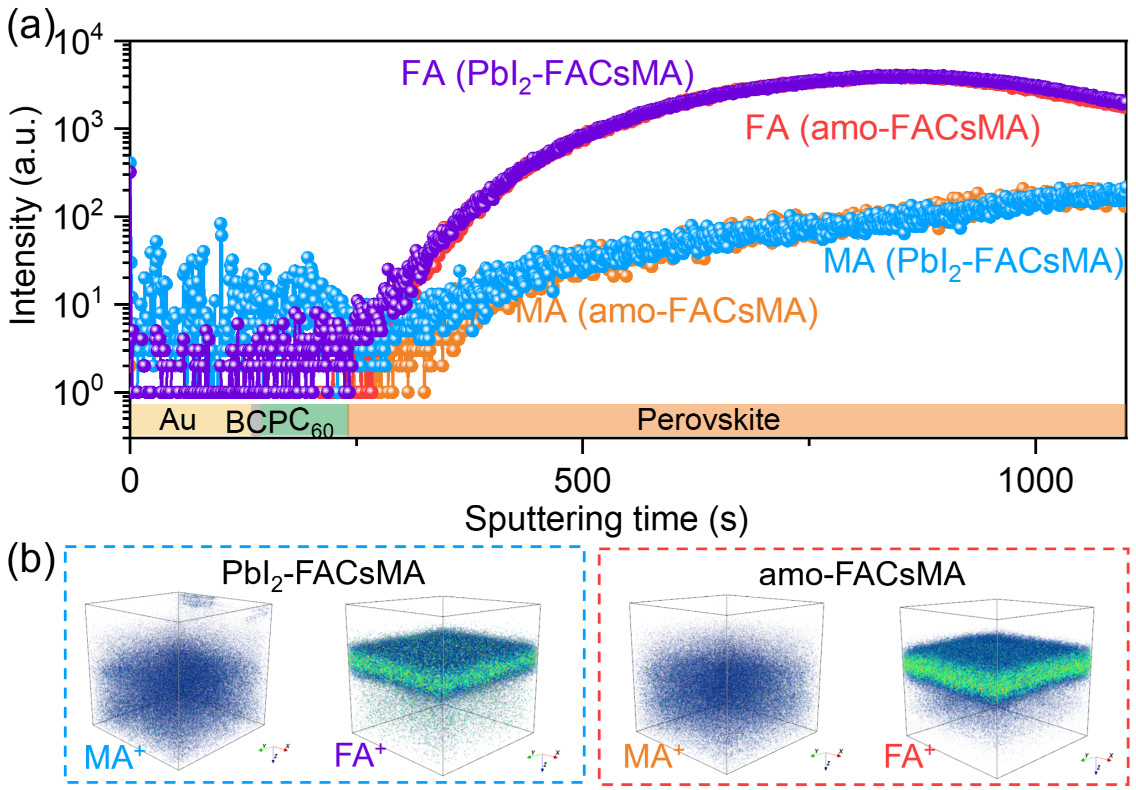

Supplementary Fig. S27 Characterization of perovskite layers in PbI_2-FACsMA and amo-FACsMA based devices before MPP tests. (a) TOF-SIMS depth profiles for the devices based on PbI_2-FACsMA and amo-FACsMA perovskites before MPP tests. Corresponding (b) 3D MA^+/FA^+ distribution in the PSCs based on PbI_2-FACsMA and amo-FACsMA perovskites before MPP tests.

Supplementary Table S1 Summary of PV parameters of J-V curves for FACsMA and amo-FACsMA PSCs (Active area: 0.09 cm^2).

		J_{sc}/mA·cm^2	V_{oc}/V	FF	η/%
FACsMA	Reverse	25.83	1.104	0.831	23.72
	Forward	25.78	1.078	0.816	22.68
amo-FACsMA	Reverse	25.84	1.184	0.858	26.27
	Forward	25.82	1.182	0.849	25.92

Supplementary Table S2 Summary of PV parameters of J-V curves for FACsMA and amo-FACsMA PSCs (Active area:1 cm^2).

		J_{sc}/mA·cm^2	V_{oc}/V	FF/%	η/%
FACsMA	Reverse	25.66	1.128	0.739	21.38
	Forward	25.67	1.118	0.663	19.03
amo-FACsMA	Reverse	25.69	1.205	0.806	24.93
	Forward	25.67	1.188	0.797	24.31

Supplementary References

1 Zhu, C. *et al.* Strain engineering in perovskite solar cells and its impacts on carrier dynamics. *Nat. Commun.* **10**, 815, (2019).

2 Xue, D.-J. *et al.* Regulating strain in perovskite thin films through charge-transport layers. *Nat. Commun.* **11**, 1514, (2020).

3 Wu, J. *et al.* Strain in perovskite solar cells: origins, impacts and regulation. *National Science Review* **8**, (2021).
