# Supplementary material for: Amorphous (lysine)2PbI2 layer enhanced perovskite photovoltaics
Source: Nat Commun. 2024 Aug 17;15:7085. doi: 10.1038/s41467-024-51551-y (PMC11330473; doi:10.1038/s41467-024-51551-y)
Supplement: Supplementary file 3 — Reporting Summary [file 41467_2024_51551_MOESM3_ESM.pdf]

nature portfolio

Corresponding author(s): Deren Yang, Yong Wang, Weihua Ning

Solar Cells Reporting Summary

Nature Portfolio wishes to improve the reproducibility of the work that we publish. This form is intended for publication with all accepted papers reporting the characterization of photovoltaic devices and provides structure for consistency and transparency in reporting. Some list items might not apply to an individual manuscript, but all fields must be completed for clarity.

For further information on Nature Research policies, including our data availability policy, see Authors & Referees.

Experimental design

Please check the following details are reported in the manuscript, and provide a brief description or explanation where applicable.

1. Dimensions

Area of the tested solar cells

☒ Yes — Figure 3a and Figure 3e.
☐ No — *Explain why this information is not reported/not relevant.*

Method used to determine the device area

☒ Yes — Characterizations part in Method.
☐ No — *Explain why this information is not reported/not relevant.*

2. Current-voltage characterization

Current density-voltage (J-V) plots in both forward and backward direction

☒ Yes — Supplementary Figure S23 and Figure S24.
☐ No

Voltage scan conditions

☒ Yes — Characterizations part in Method.
☐ No — *Explain why this information is not reported/not relevant.*

Test environment

☒ Yes — Characterizations part in Method.
☐ No — *Explain why this information is not reported/not relevant.*

Protocol for preconditioning of the device before its characterization

☒ Yes — Characterizations part in Method.
☐ No — *Explain why this information is not reported/not relevant.*

Stability of the J-V characteristic

☒ Yes — Figure 3d and Figure 4b-4c.
☐ No — *Explain why this information is not reported/not relevant.*

3. Hysteresis or any other unusual behaviour

Description of the unusual behaviour observed during the characterization

☒ Yes — The device showed negligible J-V hysteresis and no unusual behaviour.
☐ No — *Explain why this information is not reported/not relevant.*

Related experimental data

☒ Yes — Supplementary Figure S23 and Figure S24.
☐ No — *Explain why this information is not reported/not relevant.*

4. Efficiency

External quantum efficiency (EQE) or incident photons to current efficiency (IPCE)

☒ Yes — Figure 3b
☐ No — *Explain why this information is not reported/not relevant.*

A comparison between the integrated response under the standard reference spectrum and the response measure under the simulator

☒ Yes — "Photovoltaic performances of the PSCs" section in the main text.
☐ No — *Explain why this information is not reported/not relevant.*

For tandem solar cells, the bias illumination and bias voltage used for each subcell

☐ Yes — *Provide a description of the measurement conditions.*
☒ No — Not tandem devices.

5. Calibration

Light source and reference cell or sensor used for the characterization

☒ Yes — Characterizations part in Method.
☐ No — *Explain why this information is not reported/not relevant.*

Confirmation that the reference cell was calibrated and certified

☒ Yes — Characterizations part in Method.
☐ No — *Explain why this information is not reported/not relevant.*

Calculation of spectral mismatch between the reference cell and the devices under test

☒ Yes — Characterizations part in Method.
☐ No — *Explain why this information is not reported/not relevant.*

6. Mask/aperture

Size of the mask/aperture used during testing

☒ Yes — Caption and text in Figure 3a and Figure 3e.
☐ No — *Explain why this information is not reported/not relevant.*

Variation of the measured short-circuit current density with the mask/aperture area

☒ Yes — The aperture area is fixed for each device.
☐ No — *Explain why this information is not reported/not relevant.*

7. Performance certification

Identity of the independent certification laboratory that confirmed the photovoltaic performance

☒ Yes — We have provided a certification efficiency.
☐ No — *Explain why this information is not reported/not relevant.*

A copy of any certificate(s)

☒ Yes — Figure S22.
☐ No — *Explain why this information is not reported/not relevant.*

8. Statistics

Number of solar cells tested

☒ Yes — 32 devices.
☐ No — *Explain why this information is not reported/not relevant.*

Statistical analysis of the device performance

☒ Yes — Figure 3c.
☐ No — *Explain why this information is not reported/not relevant.*

9. Long-term stability analysis

Type of analysis, bias conditions and environmental conditions

☒ Yes — Figure 4a and Figure 4b. Text in "Shelf-life and operational stability of the PSCs" part.
☐ No — *Explain why this information is not reported/not relevant.*
